# Engineering and characterization of human β-defensin-3 and its analogues and microcin J25 peptides against *Mannheimia haemolytica* and bovine neutrophils

**DOI:** 10.1186/s13567-021-00956-4

**Published:** 2021-06-10

**Authors:** Harpreet Dhingra, Kamaljit Kaur, Baljit Singh

**Affiliations:** 1 Department of Veterinary Biomedical Science, Western College of Veterinary Medicine, University of Saskatchewan, Saskatoon, SK S7N 5B4 Canada; 2 Chapman University School of Pharmacy (CUSP), Harry and Diane Rinker Health Science Campus, Chapman University, Irvine, CA 92618-1908 USA

**Keywords:** HBD3, Microcin J25, Cationic anti-microbial peptides, Solid phase peptide synthesis (SPPS), Bovine neutrophils, Chemotaxis

## Abstract

*Mannheimia haemolytica*-induced bovine respiratory disease causes loss of millions of dollars to Canadian cattle industry. Current antimicrobials are proving to be ineffective and leave residues in meat. Antimicrobial peptides (AMPs) may be effective against *M. haemolytica* while minimizing the risk of drug residues. Cationic AMPs can kill bacteria through interactions with the anionic bacterial membrane. Human β-Defensin 3 (HBD3) and microcin J25 (MccJ25) are AMPs with potent activity against many Gram-negative bacteria. We tested the microbicidal activity of wild-type HBD3, three HBD3 peptide analogues (28 amino acid, 20AA, and 10AA) derived from the sequence of natural HBD3, and MccJ25 in vitro against *M. haemolytica*. Three C-terminal analogues of HBD3 with all cysteines replaced with valines were manually synthesized using solid phase peptide synthesis. Since AMPs can act as chemoattractant we tested the chemotactic effect of HBD3, 28AA, 20AA, and 10AA peptides on bovine neutrophils in Boyden chamber. Minimum bactericidal concentration (MBC) assay showed that *M. haemolytica* was intermediately sensitive to HBD3, 28AA and 20AA analogues with an MBC of 50 µg/mL. The 10AA analogue had MBC 6.3 µg/mL which is likely a result of lower final inoculum size. MccJ25 didn’t have significant bactericidal effect below an MBC < 100 µg/mL. Bovine neutrophils showed chemotaxis towards HBD3 and 20AA peptides (*P* < 0.05) but not towards 28AA analogue. Co-incubation of neutrophils with any of the peptides did not affect their chemotaxis towards N-formyl-l-methionyl-l-leucyl-phenylalanine (fMLP). The data show that these peptides are effective against *M. haemolytica* and are chemotactic for neutrophils in vitro.

**Supplementary Information:**

The online version contains supplementary material available at 10.1186/s13567-021-00956-4.

## Introduction

Cattle industry is a major contributor to global economy and is expected to play a critical role in meeting nutritional needs of expanding middle classes in emerging economies such as China and India. For example, Canada has nearly 83 000 cattle farms and ranches and this sector contributes upwards of $20 billion a year to Canadian economy [1]. The beef and dairy cattle industry suffer significant economic losses due to *Mannheimia haemolytica-*induced Bovine respiratory disease (BRD) [2]. *M. haemolytica*, a Gram-negative coccobacillus that causes pneumonia in cattle, resides in the nasopharynx of cattle in a commensal relationship, but stress and viral infections compromise host defenses giving *M. haemolytica* an opportunity to invade lungs and cause infection [3]. A variety of vaccines and antibiotics against *M. haemolytica* are available but control and prevention are still a problem and due to which approximately $1 billion per year are lost by the US cattle industry [2–6] with similar relative losses in other countries such as Australia and Canada [7]. There is a growing evidence of development of resistance in *M. haemolytica* against commonly used antimicrobials and a call to take quick steps to develop new and effect anti-microbials to combat this pathogen [8, 9]. Problems such as antibiotic resistance, antibiotic residue in meat and an inadequacy of vaccines push for the search of alternative treatments.

In this context, antimicrobial peptides (AMPs) have received significant attention as alternatives to antibiotics [10, 11]. Human β-Defensin 3 (HBD3) has been isolated from airways surface fluid from patients with psoriasis, suggesting it may play a role in fighting local infection [12]. Recent work by the Caswell laboratory has shown that treatment with naturally expressed Tracheal Antimicrobial Peptide, a defensin, neither killed *M. haemolytica* nor prevented the development of lung disease [13, 14]. Coupled with the fact that *M. haemolytica*, in normal condition, is prevented from entering into the lungs by the host’s innate defense mechanism which includes the bovine defensins [3], it was thought that HBD3 peptide might be effective in *M. haemolytica*-induced pneumonia. Microcins are another class of AMPs which are produced by bacteria such as *E. coli*. Microcins inhibit the growth of many pathogenic Gram-negative bacteria with Minimum inhibitory concentration (MIC), in the nanomolar range [11, 15–20]. Specifically, MicrocinJ25 (MccJ25) is a peptide comprised of 21 amino acids and is active against many Gram-negative bacteria such as *Escherichia coli* and *Salmonella* [21]. Mccj25 is stable in the presence of many proteolytic enzymes because of its unique lasso like structure [11]. Considering the economic impact of *M. haemolytica* infections in cattle and lack of effective anti-microbial peptides to replace antibiotics, we hypothesize that HBD3 and MccJ25 might prove as potential candidates for application in the treatment of BRD. The objective of the current study was to design, synthesize, purify and evaluate the antimicrobial activity of HBD3, its analogues, and recombinant MccJ25 against *M. haemolytica*.

## Materials and methods

### Expression and purification of microcin J25

Wild-type Microcin J25 (MccJ25) was used in this study as formation of the lasso ring is not possible synthetically. The peptide was expressed using MccJ25 biosynthetic gene cluster carrying plasmid pTUC202 (a gift from Rutgers University USA) in competent *E. coli* MC4100 cells [25]. It was grown in 2L M9 minimal media for 18 h at 37 °C. The culture supernatant was obtained by centrifugation at 4000 *g* for 15 min, and then subjected to two successive purification steps. First, it was applied on the flex column filled with XAD16 resin (Sigma-Aldrich, St. Louis, USA). Two successive elution steps were performed with (30:70, v/v) and (80:20, v/v) methanol/water mixtures. MccJ25 is eluted in the (80:20, v/v) methanol/water mixture. MccJ25 was purified using reversed-phase (RP) HPLC (Varian Prostar 210, SpectraLab Scientific Inc. Markham, Canada). The purity of the peptides was confirmed by MALDI-TOF mass spectrometry.

### Synthesis and purification of HBD3 analogues

HBD3 fragments were synthesized manually on 2-chlorotrityl chloride resin (0.2 mmol, 1 mmol/g) following the standard Fmoc solid phase peptide synthesis (SPPS) [22]. The first amino acid was coupled using DIPEA for 5 h. Further amino acids were coupled at twofold excess using HCTU/HOBT/NMM as activating mixture in DMF. Amino acid coupling was performed for 3 h at room temperature to ensure the completeness of the reaction, followed by Fmoc group deprotection using piperidine in DMF. These two reactions were repeated until assembly of the peptide was complete. A Kaiser test was performed after coupling of each amino acid. After completion of the synthesis, peptides were cleaved from the resin and all protecting groups were removed using cleavage mixture at room temperature. Cleaved peptide was concentrated by rotary evaporation. Cold ether was added to precipitate the peptide and crude peptide was isolated after centrifugation. Crude peptides were dissolved in water and purified using RP-HPLC (Varian Prostar 201). The purity of the peptides was confirmed by RP-HPLC and MALDI-TOF mass spectrometry.

### Antimicrobial activity testing of peptides

Wild-type HBD3, its analogues, and MccJ25 were tested against *M. haemolytica-A1* using optical density method and colony count assay. Briefly, an aliquot (5 µL) from bacteria suspension stored at −80 °C was put in 5 mL BHI broth at 37 °C for 18 h. An aliquot (10 µL) from this overnight culture was added to fresh BHI broth and incubated at 37 °C for a further 5 h to obtain mid-log phase bacteria. The culture was then centrifuged for 10 min at 800 *g* at 4 °C. The supernatant was discarded and the bacterial pellet was resuspended in cold, sterile sodium phosphate buffer (SPB) and washed again at 800*g* for 10 min at 4 °C. The pellet was resuspended in 5 mL SPB. To calculate the number of CFU/mL in the 5 h culture, six successive tenfold dilutions were made. From the last dilution 100 µL was plated in duplicate on petri dishes containing BHI agar and incubated for 24 h. After 24 h the colonies were counted in the two petri dishes and then averaged. Total number of colonies was the average number of colonies multiplied by the dilution factor.

All assays were carried out in sterile 96-well polypropylene flat-bottomed plates using a broth microdilution method. Two-fold serial dilutions of peptide were made in SPB, and 50 µL of each concentration of peptide was added to the wells of the assay plate. Fifty µL (25 000 cells) of the bacterial suspension adjusted to 5 × 10^5^ cfu/mL in incubation media was prepared by adding 200 µL of BHI broth in 6.8 mL SPB and added to each well. Positive control wells contained 50 µL of the bacteria with 50 µL of 64 µg/mL ampicillin and negative control wells contained 50 µL of SPB and 50 µL bacterial suspension. Sterility control well contained 100 µL BHI broth to test and ensure that broth was not contaminated.

The plates were sealed with aluminum foil and were incubated at 37 °C for 2 h then 50 µL of the contents of the well were pipetted on BHI agar plates and incubated for 24 h. The bactericidal activity was expressed as the MBC which is the concentration at which 99.9% of the colonies are killed and the Lethal dose 50 (LD_50_) at which 50% or more bacteria are killed [23–25]. All experiments were run in triplicates.

### Polymorphonuclear cell isolation and chemotaxis assay

#### Polymorphonuclear cell isolation

Neutrophils were isolated from cattle using established methods and approved by the University of Saskatchewan’s Committee on Animal Care [26]. For each individual animal, blood was pooled into 50 mL tubes and diluted with an equal volume of PBS then 12.5 mL of the diluted blood was layered over 10 mL of Ficoll Paque PLUS (Sigma-Aldrich, St. Louis, USA) while taking care to preserve the interface between the two liquids. Following centrifugation at 400 *g* for 30 min at 20 °C with the brake turned off, the lymphocyte layer was discarded along with Ficoll and plasma. The pellet was washed in 20 mL PBS with centrifugation at 500 *g* with low brake for 10 min. The supernatant was discarded and the pellet was suspended in equal volume of sterile water and gently mixed for 20 s followed by addition of equal volume of 1.8% NaCl to restore tonicity. The mixture was centrifuged at 500 *g* for 10 min and followed by another washing and centrifugation at 4 °C. The resulting neutrophil pellet was resuspended in PBS. Bovine neutrophils, collected in the above manner had more than 92% purity, and their viability was greater than 95% based on Trypan blue exclusion assay. Therefore, we use the term neutrophils in the manuscript.

#### Chemotaxis assay

We used fMLP (114 nm) as the chemoattractant. Approximately 25 μL of peptides (50 μg/mL) was put into lower compartment wells of a 48-well Boyden Chamber. After the fMLP loading, the polycarbonate membrane filter (pore size 5 μM), with the shiny side up, was placed over the lower chamber. Next, silicone gasket was placed on the membrane, then the upper chamber was put on top of the gasket and the lug nuts firmly secured. Cell suspension (1 × 10^6^ cells) was then placed over control wells and N/P wells (neutrophil at top and peptide in the bottom wells). The whole assembly was then incubated at 37 °C in humidified air with 5% CO_2_ for 20 min. After the incubation, the Boyden chamber was disassembled and the membrane carefully held with the bulldog clamp and the cells on the upper shiny surface scraped with the wiper blade. Next, the filter membrane was stained with the Diff-Quick and mounted on glass slide with the bottom side up. The cells within the filter pores were then counted in five random fields under light microscope at 40×. The results are presented as the number of migrated neutrophils per microscopic field.

### Migration inhibition assay

Since peptides can be immunomodulatory, migration inhibition was carried out to determine if the peptides inhibit neutrophil migration. This assay was performed in the same manner as the chemotaxis assay with the only difference being that peptide and neutrophils were incubated together on the top of the filter; 25 μL of 100 μg/mL peptide and 25 μL (2 × 10^6^ cells) of neutrophils were put together. In the lower wells 25 μL of 114 nM fMLP (chemoattractant) was placed followed by the staining and counting of the cells as described above.

### Statistical analysis

Data from susceptibility testing assay was analyzed using the non-parametric statistics (using ranked data), because the data were variable among groups most likely due to small sample size. The comparisons were done using contrasts (1 compared to 2, 2 compared to 3, and so on), given that we expected a decrease in colony count with increasing concentrations. Groups were considered statistically significant if the *P*-value was less than 0.05. A One-way ANOVA followed by Dunnets Multiple Comparisons test was performed on data obtained from the chemotaxis and migration inhibition assay. Significance was recorded when *P* < 0.05. Computer software GraphPad Prism 6 and SPSS (GraphPad Software, San Diego, USA) for migration and susceptibly assays, respectively, were used.

## Results

### Peptide design and synthesis

**Figure 1:**
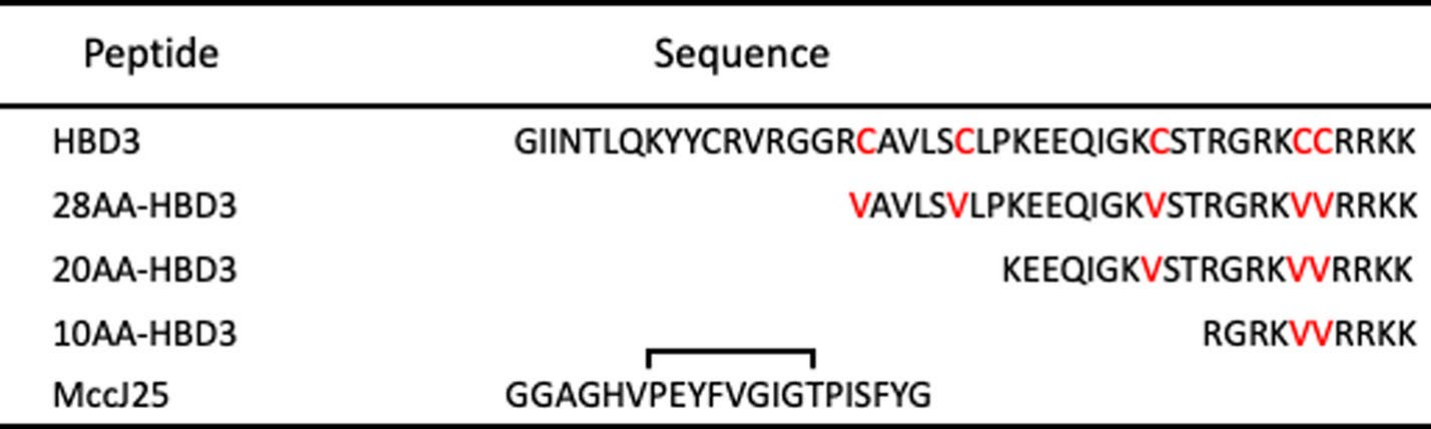
**Amino acid sequences of HBD3, and the three HBD3 fragments.** Substitution of cysteine residues with valine in the fragments is shown in red.

Five peptides, HBD3, 28AA HBD3, 20AA HBD3, 10AA HBD3, and Microcin J25 (MccJ25) were evaluated in this study. Because wild type HBD3 is commercially available, we purchased it at 95% purity (AnaSpec Inc.). The amino acid sequences of the HBD3 and its analogues and MccJ25 studied are shown in Figure 1.

Approximately 3 mg Wild-type MccJ25 was obtained by over expression protocol already established by Soudy et al. [27]. After purification by flex column the crude MccJ25 was finally purified with the C-18 RP-HPLC, at a flow rate of 2 mL/min 55–80% methanol/water mixture in 55 min. Various fractions at different time points were obtained. Fraction containing the desired mass for MccJ25 eluted at 20 min on the RP-HPLC column (Additional File 1). The calculated mass for MccJ25 was 2107 MW and the mass was determined to be [M + H]^+^ 2107.7 (Additional File 1). The elute from desired peak was collected, pooled and lyophilized. The yield or the concentration of MccJ25 was measured using UV–Vis spectrophotometer at 278 nm.

### SPPS synthesis of HBD3 analogues

Three C-terminal HBD3 analogues were designed as short fragments of wtHBD3 with different amino acid chain lengths (Figure 1). In each analogue, cysteine was replaced with valine to remove di-sulfide linkages and render the fragment linear and more stable. Standard SPPS with Fmoc chemistry was used for the synthesis of the three analogues. Manual SPPS was carried out in a reaction vessel starting with the shorter 10AA analogue. Amino acid chain was built on 2-chlorotrityl resin in the reaction vessel. The proper peptide chain elongation was ensured with Ninhydrin test after every coupling step and test cleavage after every third amino acid. After building 10AA amino acid chain, the peptide was cleaved from the solid support or resin. A treatment with 95% TFA, 5% tri-isopropylsilane in water, for 2 h at room temperature with continuous shaking of the vessel was used to simultaneously de-protect the side chains and cleave the crude peptide from the resin. On a semi-preparative RP-HPLC column using gradient elution, 22 mg of the crude 10AA analogue was purified. A gradient method with 10–35% acetonitrile/water in 60 min with a flow rate of 1.3 mL/min was used on the RP-HPLC for the purification of crude synthetic peptide 10 AA.

Peaks eluted at 10.9–11.2, 13.4–14.5, 17–18, 26–27, 27–28, and 33–34 min were collected. Mass spec, through MALDI-TOF with α-cyano-4-hydroxycinnamic acid (HCCA) as a matrix, for all of the elutions was taken and elution at peak 13.4–14.5 had the correct mass. Calculated mass for 10AA was 1283 and the mass found was [M + H]^+^ 1282.6. (Additional file 7).

20AA analogue was synthesized in a manner similar to 10AA analogue. Beyond the addition of tenth amino acid, ninhydrin test and test cleavage were performed after every third coupling. Double coupling was performed for the last four amino acids, because of the positive ninhydrin test after single coupling. The purification scheme used for 20AA analogue was the same as the 10AA analogue. Peaks eluted at 13–14.5, 23–25, 29–30, 35–36.5, and 51.5–53 min were collected. Similarly, the MALDI-TOF mass spectrum for all of the elutions was taken and the correct mass was found in elution at peak 35–36.5 (Additional file 3). Calculated mass for 20AA was 2384 and the mass found was [M + H]^+^ 2383.3 (Additional file 3). However, complete resolution between 19AA analogue and 20AA analogue could not be achieved. Several other HPLC gradient were tried to achieve resolution of 20AA analogue from other truncated peptides but best results were achieved with 10–35% acetonitrile/water in 60 min with a flow rate of 1.3 mL/min scheme. Nonetheless, the major component from the eluate was 20AA analogue and 19AA analogue was only a small fraction. Overall 24–28 mg of purified peptide was obtained.

The synthesis of 30AA, linear with all cysteines mutated with valine, was attempted. However, the chain could be elongated only up to 28 AA analogue as identified by mass spec of crude peptide showing a mass of 3386.4 [M + H]^+^ (Calculated 3387) (Additional file 4). The coupling of arginine to the 28th amino acid valine could not be achieved even with three consecutive couplings. Since the manual synthesis of such a long amino acid chain is usually fraught with many pitfalls, the elongation of a 28 amino acid chain was a satisfactory result, because the appropriate balance of positive charge and hydrophobicity was attained.

### Microbicidal effect of peptides

**Figure 2:**
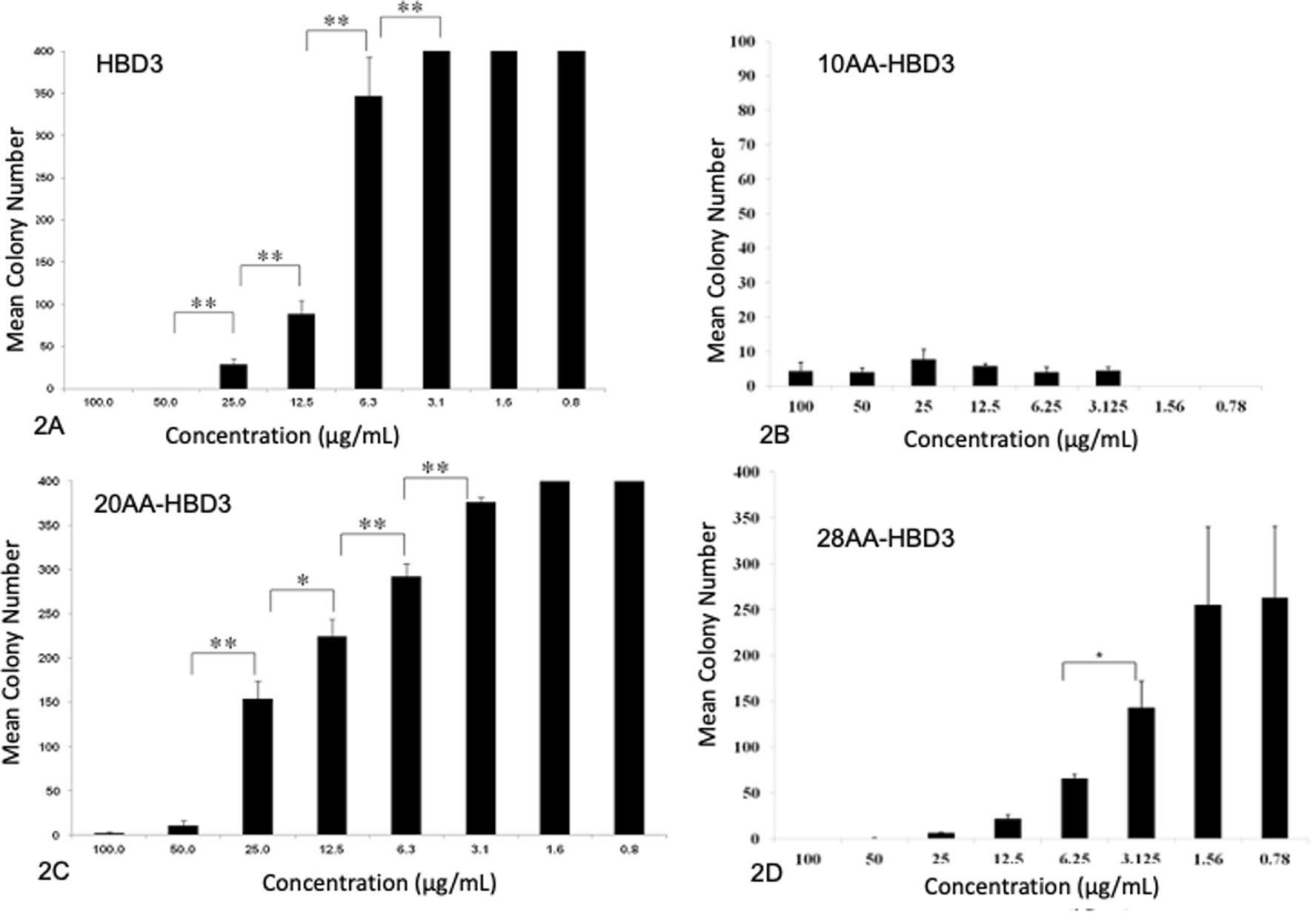
**Comparisons between microbicidal effects of eight different concentrations of HBD3 and HBD analogues.** Comparisons were performed using contrasts for example 100.0 μg/mL was compared to 50.0 μg/mL, 25.0 μg/mL compared to 12.5 μg/mL employing non-parametric statistics (ranked data). Statistical significance between two concentrations is indicated by *P < 0.05 and **< 0.01. The data are represented as Mean ± SEM.

**Figure 3:**
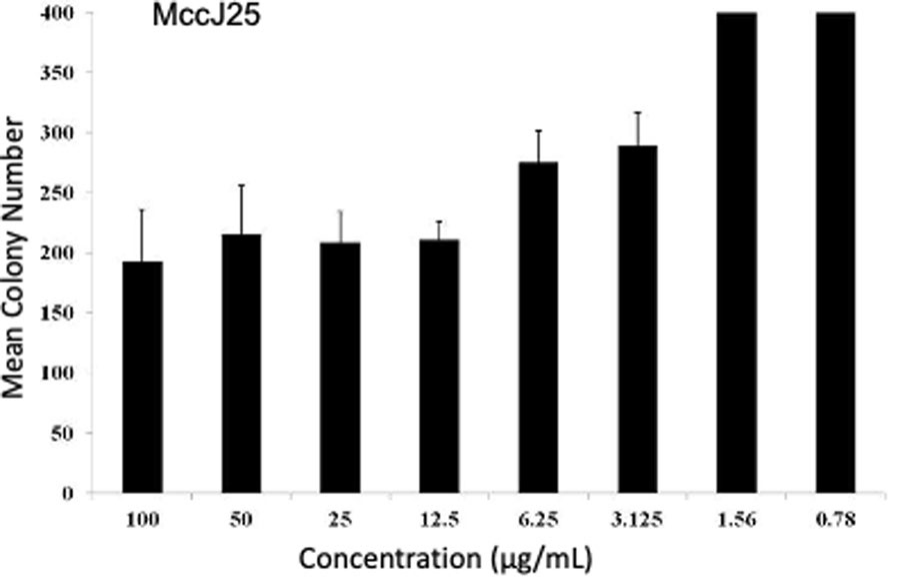
**Comparisons between microbicidal effects of eight different concentrations of MccJ25.** Comparisons were performed using contrasts for example 100.0 μg/mL was compared to 50.0 μg/mL, 25.0 μg/mL compared to 12.5 μg/mL employing non-parametric statistics (ranked data). Statistical significance between two concentrations is indicated by *P < 0.05 and **< 0.01. The data are represented as Mean ± SEM.

Peptides HBD3, its analogues and Mccj25 were assessed for Minimum bactericidal concentration (MBC). MBC is described as the lowest concentration of each drug that resulted in a 99.9% reduction in CFU of the initial inoculum. All the peptides have shown intermediate to potent killing activity towards *M. haemolytica* (Figures 2, 3). The final inoculum size should be 5 × 10^5^ cfu/mL, but varied between 1 × 10^5^ and 1 × 10^6^ cfu/mL and is acceptable as per M7-A7 guidelines. However, some published studies have taken final inoculum in the range of 10^4^–10^5^ cfu/mL [28].

The comparison of antibacterial activity at different concentrations of HBD3 revealed that HBD3 was equally active at 100.0 µg/mL and 50.0 µg/mL. However, the concentrations 50 µg/mL and 25.0 µg/mL, 25.0 µg/mL and 12.5 µg/mL, 12.5 µg/mL and 6.3 µg/mL, and 6.3 µg/mL and 3.1 µg/mL were statistically different. The concentrations 3.1 µg/mL and 0.8 µg/mL were not active and there was no statistical difference between them (Additional file 7 and Figure 2A).

The activity of 10AA at all the concentrations was similar without any statistical difference. It is worth noting that this is observed only with 10AA analogue which is very likely due to a smaller inoculum size (10^4^ cfu/mL) used (Additional file 8 and Figure 2B). Similar to HBD3, 20AA analogue was equally active at both 50.0 µg/mL and 100.0 µg/mL. However, the concentrations 50.0 µg/mL and 25.0 µg/mL, 25.0 µg/mL and 12.5 µg/mL, 12.5 µg/mL and 6.3 µg/mL, and 6.3 µg/mL and 3.1 µg/mL were statistically different from each other. Again, concentrations 3.1 µg/mL and 0.8 µg/mL were not active and there was no statistical difference among them (Additional file 9 and Figure 2C).

The antibacterial effect of 28AA analogue’s concentrations 100.0 µg/mL to 6.3 µg/mL was statistically similar as was the antibacterial effect for the concentrations 3.1–0.8 µg/mL. The only difference was observed between 3.1 and 6.3 µg/mL (Additional file 10 and Figure 2D and Additional file 3). Additional file 3 shows a clear dose response when *M. haemolytica* was treated with crude 28AA analogue. The number of colony forming units progressively increased with decreasing peptide concentration. The MBC and LD_50_ of all the peptides have been compared (Table 1). While HBD3 and HBD3 20AA analogues have equal MBC values of 50 µg/mL, MBC value for HBD3 28AA analogue was the lowest at 12.5 µg/mL, and MccJ25 had highest MBC value of 100 µg/mL (Additional file 10). Nearly comparable LD_50_ values do not effectively discriminate different peptides. However, the value of LD_50_ is apparent in the fact that it shows MccJ25 kills half of the bacterial population at 6.3 µg/mL.

**Table 1:** **Comparisons of MBC and LD**_**50**_ **of the peptides**

Peptide	MBC (μg/mL)	LD_50_ (μg/mL)
HBD3 10AA analogue	6.3	≥ 3.1
HBD3 20AA analogue	50.0	3.1
HBD3 28AA analogue	12.5	3.1
HBD3	50.0	6.3
MccJ25	> 100.0	6.3

MBC (Minimum Bactericidal Concentration) is the concentration of antibiotic at which 99.9% of the CFU in the final inoculum are killed and LD_50_ is the lethal dose for ≥ 50% of bacteria.

MccJ25 exhibited microbicidal effect; however, the reduction in colony count was much less in comparison to other peptides Different concentrations of MccJ25 were compared for antibacterial activity against *M. haemolytica* but no statistically significant difference was observed (Additional file 11 and Figure 3).

### Chemotaxis assay

**Figure 4:**
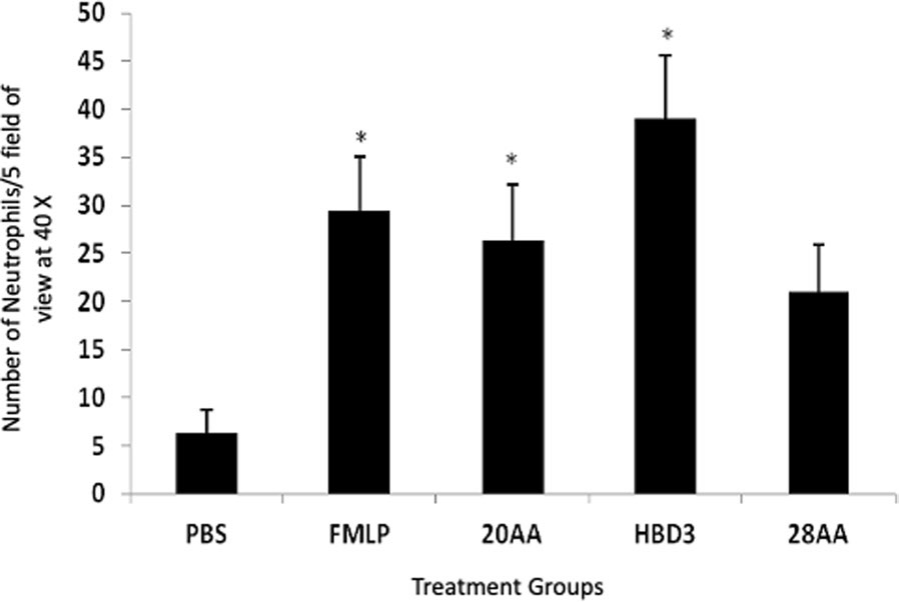
**Chemotaxis assay of the peptides against bovine neutrophils.** Number of neutrophils that migrated in response to HBD3, HBD3 20AA analogue and, HBD3 28AA analogue after 20 min incubation at 37 °C. The three peptides were used at a concentration of 50.0 μg/mL. fMLP is the positive control. Mean ± SEM of three independent experiments. One way ANOVA was followed by Dunnett’s multiple comparison test (*P* ≤ 0.0036). Asterisks show significant difference from PBS (Negative control) *P < 0.05.

HBD3 is chemotactic for human neutrophils [29]. The chemotaxis of bovine neutrophils in response to HBD3, HBD3 20AA analogue, and HBD3 28AA analogue at 50.0 μg/mL was studied. Neutrophil migration in response to HBD3 and 20AA analogue was found to be statistically significant when compared with PBS (Negative control), but the difference between PBS and 28AA was not significant (Figure 4). The result shows that HBD3 and HBD3 20AA analogue were chemotactic for bovine neutrophils at 50.0 μg/mL where as 28AA analogue was not.

### Migration inhibition assay

**Figure 5:**
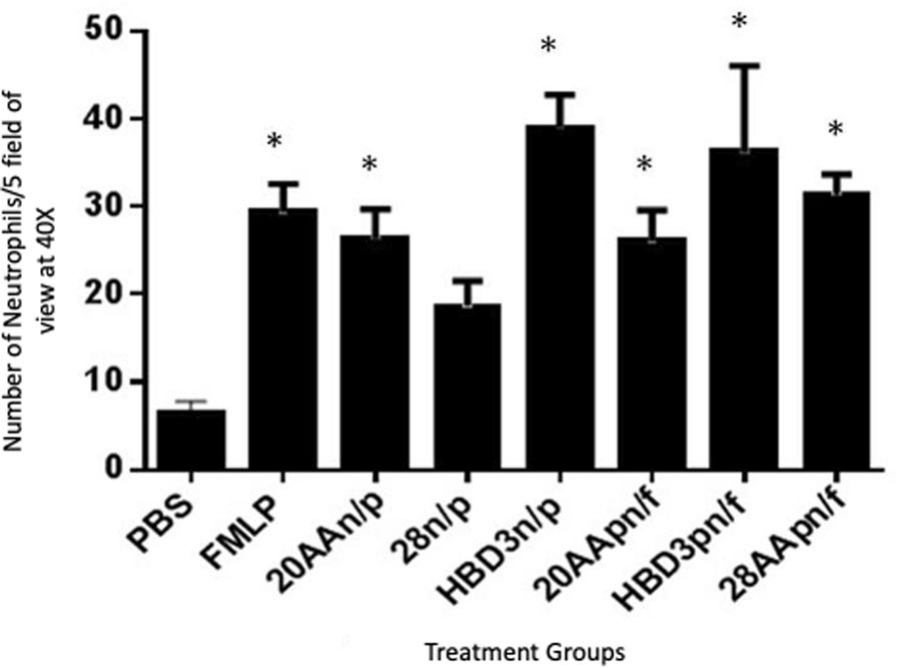
**Effect of peptides on neutrophil migration towards fMLP.** Number of neutrophils that migrated towards fMLP (114 nM) after 20 min incubation at 37 °C. Neutrophils were treated with 50.0 μg/mL HBD3, HBD3 20AA analogue and, HBD3 28AA analogue. pn/f stands for peptide and neutrophils together in the upper well and fmlp in the bottom during 20 min incubation. fMLP is the positive control. Mean ± SEM of three independent experiments. One way ANOVA was followed by Dunnett’s multiple comparison test (*P* ≤ 0.0036). Asterisks show significant difference from PBS (Negative control) *P < 0.05.

Bovine neutrophils were treated with 50.0 μg/mL of HBD3, HBD3 20AA analogue, and HBD3 28AA analogue and the effect of peptides on neutrophil migration towards fMLP was studied. Neutrophil migration towards fMLP when neutrophils were incubated with HBD3, HBD3 20AA analogue, and HBD3 28AA analogues was statistically different from the PBS, which shows that none of the peptides inhibited migration of neutrophils towards fMLP (Figure 5).

## Discussion

To our knowledge, this is the first study to show that based on MBC and LD_50_ data HBD3 and its analogues and MccJ25 peptides kill *M. haemolytica* in vitro. In addition, the peptides are chemotactic for the bovine neutrophils. These data are important considering the economic impact of morbidity and mortality associated with BRD caused by *M. hemotlyica*. The development and use of antimicrobial peptides may also provide us with tools to reduce the use of antibiotics and thus reduce the threat of anti-microbial resistance and their residues in meat.

HBD3 and MccJ25 were selected as potential candidates. Wild-type HBD3 with purity ≥ 95% was commercially obtained, whereas MccJ25 was expressed in *E. coli* followed by purification and characterization. The data contained in this study show synthesis and characterization of three analogues of HBD3. We successfully synthesized HBD3 analogues (Figure 1) namely 10AA, 20AA but couldn’t synthesize 30AA using Fmoc-SPPS. During SPPS, the -amino acid chain could be elongated up to only 28AA as confirmed by MALDI-TOF even after repeated coupling. Mass spectrometry of 28AA crude peptide shows other peaks along with 28AA analogue peak suggesting impurities in the crude peptide. The crude peptides (MccJ25, 10AA and 20AA analogue) were purified by RP-HPLC and then by MALDI-TOF mass spectrometry to confirm mass of the crude and pure peptides.

In vitro susceptibility tests for cationic peptides are challenging to perform because polycationic peptides tend to precipitate and bind to the anionic surface of bacterial cells and plastic surface. There are data on the comparison of two methods for evaluating the in vitro antimicrobial activities of cationic AMP’s [30]. The comparative data showed that NCCLS protocol gave MICs and MBCs values that were four times higher than the method proposed by Hancock [30]. We used the 96-well plates for antimicrobial evaluation to reduce the possibility of false higher MBCs and MICs as apparent in the results of Giacometti and the results of our preliminary MIC experiment (data not shown). However, we used the NCCLS definition of MBC which is the lowest concentration of each drug that resulted in a 99.9% reduction in CFU of the initial inoculum whereas the Hancock method defines the MBC as the lowest concentration of each drug that prevented any residual colony formation. In addition to the MBC, we also carried out the MIC assays. This classification of peptide potency however is arbitrary, “a strain was defined as sensitive to the peptide if MBC levels were < 10 μg/mL, as intermediately sensitive if MBC levels were 10–100 μg/mL, or as resistant at MBC levels of > 100 μg/mL” [28].

In our study, HBD3 was highly effective in killing *M. haemolytica* with MBC value of 50 μg/mL. The testing of the bactericidal activity of the HBD3 against 28 species and 55 strains of Gram-positive cocci and Gram-negative fermentative and non-fermentative rods showed it be intermediately or highly effective [28]. However, MBC value in our study is higher when compared to the data from Sahly et al. in which only three strains out of 55 tested showed intermediate susceptibility (MBC value of 50.0 μg/mL), whereas rest of the strains were highly sensitive to HBD3 with MBC’s ranging from 0.1 to 6.3 μg/mL [28]. The reason for lower MBC’s in their study could be attributed to use of a lower final bacterial inoculum of 10^4^ to 10^5^/mL [28] compared 10^5^ to 10^6^/mL used in our study. This phenomenon of lower MBC corresponding with low final inoculum size was also observed in our data from 10AA analogue. We tested various concentrations of this analogue with final inoculum size of 10^4^ cells/mL of *M. haemolytica* and this gave us MBC of 6.3 μg/mL.

HBD3 analogues showed intermediate activity against *M. hemolytica*. 20AA analogue was active against *M. haemolytica* in the same concentration as the wild type HBD3, after averaging results from three different experiments, in duplicates, they showed equivalent MBC values. 20 AA analogue’s potent activity is likely attributable to its high positive charge (+9). HBD has a higher positive charge of +11 but showed anti-microbial activity similar to that of 20AA analogue. Therefore, it will be interesting to replace a couple of positively charged basic polar amino acids such as arginine or lysine with valine, a neutral nonpolar amino acid, to the analogue more hydrophobic and increasingly selective for bacterial membranes. This argument is further supported by the fact that 10 AA analogue with +7 charge displayed excellent activity against *M. haemolytica* with 6.3 μg/mL MBC when used with a small inoculum. The testing of this analogue with a higher bacterial inoculum, similar to other peptides, would have further bolstered our results. Nevertheless, MBC of 10AA analogue was lower than both HBD3 and 20AA analogue and therefore we may be able extrapolate this result for higher inoculum size.

Zhou et al. made eight C-terminal 10AA analogues of HBD3 replacing cysteines with either Valine, Tryptophan or Tyrosine. Out of these, Valine was easier to build via SPPS synthesis and was found to be most active and it also didn’t affect antimicrobial activity against bacteria such as *Pseudomonas aeruginosa* [31]. Because cysteine and methionine are prone to oxidation, they are often replaced by hydrophobic residues such as valine [32]. They also made a dimer of Valine 10AA analogue, which turned out to be the most potent analogue. Instead of making a dimer of 10AA analogue, we chose to elongate the peptide chain up to 20 and 28 AA with mutation of all Cysteines with Valines. Both 20AA and 28AA analogues were active with 28AA peptide showing better activity than wild-type HBD3 [31]. These researchers further tested their peptides against *Pseudomonas aeruginosa*, a Gram −ve bacteria, in the same concentration range as ours. Comparing their study with ours, we found that the 10AA analogue had similar MBC values (50.0 μg/mL) for both *Pseudomonas aeruginosa* and *M. haemolytica*. However, their dimer was more potent than our 20AA analogue. The in vitro data also shows that removal of disulfide bonds does not affect the antimicrobial potency of the peptides as shown previously [29]. However, in vivo studies are required to further establish this observation.

Studies have shown that some C-terminal (R36-K45) analogues of HBD-3 can non-covalently dimerize to acquire a defined structure in conditions mimicking biological systems or on the lipid bilayer [31, 33]. Elucidating the underlying physico-chemical properties was beyond the scope of this study. However, we can speculate about the 3D structure of 20AA analogue based on the nearly comparable MBC results of 20AA analogue with wtHBD3, and findings of the previous studies performed with NMR, fluorescence correlation spectroscopy and molecular dynamics simulation techniques [33]. It is possible that the 20AA analogue, dimerized both in aqueous solution and on the membrane surfaces, which tend to localize the positive charge density which may account for its bactericidal efficacy. Alternatively, the 20AA analogue would just remain as a linear monomer and its potent activity results from increased flexibility due to a loss of secondary structure. It also is possible that the activity of 20AA analogue could be due to truncated peptide (19AA) that coeluted during purification of the peptide and was used.

MccJ25 kills bacteria by inhibiting the RNA Polymerase (RNAP) but this microcin peptide has to enter the bacterial cell to accomplish this action. The MccJ5 sensitive strains have the transporter proteins such as outer-membrane protein FhuA and the inner membrane protein SbmA to facilitate the transport of the antimicrobial peptides into the cells [34, 35]. There are some data on the characterization of outer membrane proteins of *M. haemolytica* [36, 37] but we don’t know whether specific membrane proteins exist to facilitate transport of MccJ25. Furthermore, MccJ25 is not effective against many bacteria of the family *Enterobacteriaceae* that are not related to the MccJ25 producing members [38]. It could be argued that MccJ25 shows a narrow action spectrum activity in vitro but could be active in vivo against resistant species [38, 39]. It has been shown that resistant bacteria become susceptible to antibiotics upon their entry into the low pH environment in the phagolysosomes of macrophages likely due to the nonspecific MccJ25 uptake into the bacterial cell through altered bacterial membrane permeability. Also, MccJ25 has been shown to cause disruption of the membrane potential [39] and hence it can be speculated that inside a macrophage *M. haemolytica* could become sensitive to MccJ25.

None of the five peptides tested by us inhibited visible growth of the *M. haemolytica* when compared with the positive control. The bactericidal nature of the cationic peptides could be the possible explanation of these results. Ampicillin at a concentration of 24 μg/mL served as negative control in our experiments and there was no growth in the negative wells. Since there were 10 000–25 000 bacteria/well and even if 0.1% of them survived and the peptide got consumed or deactivated over next few hours and media was still available for their growth. It is very likely that bacteria resumed growth to give false negatives. To ascertain the real cause, we need to do additional time-kill studies as well as testing against other serotypes of *M. haemolytica*.

Anti-microbial peptides kill microbes and have immunomodulatory functions such as modulating recruitment of neutrophils [40]. Defensins such as HBD2 but not HBD1 have been shown to be chemotactic for human neutrophils pre-treated with TNF-α but not normal in vitro [35]. Our data show HBD3 and its 20AA analogue to be chemotactic to control untreated bovine neutrophils. The difference in results from our experiments and those obtained previously may be reflective of species differences. Furthermore, we used the peptides at 50.0 μg/mL and compared to concentrations of 0–10 μg/mL in previous studies [41]. To elucidate the chemotactic behavior of 20AA analogue it must be tested against a wide concentration range and also for neutrophils from other species. It was earlier reported that cysteine mutated analogues of HBD3 might lose their chemotactic activity. It might be possible that bovine neutrophils are generally responsive to the Cysteine mutated HBD3 analogues. The data by Taylor et al. demonstrated the indispensability of three cysteine disulfide bridges for chemotactic activity [42]. They showed that HBD3 analogues lacking disulfide bridges retained chemoattractant capability. HBD3 analogue which had cysteines replaced with alanines was devoid of any chemotactic activity. However, the chemotactic activity was not lost when the fifth cysteine was retained and other five were replaced. These anomalous results could be due to variation in environmental conditions or different cell species used [42]. The chemotaxis in 28AA analogue well and PBS well was statistically not different. This anomaly can be accounted for if we take into consideration that 28AA was in crude form and had contained a few truncated peptides. Lastly, the treatment of neutrophils with any of peptides did not affect their migration towards fMLP. Nevertheless, there is a need for additional experiments to understand the relationship between structure and function of the HBD3 and its linear analogues with their chemotactic property.

Taken together, we report the engineering and synthesis of HBD analogues and MccJ25 and report their efficacy in killing of *M. haemolytica* and chemotactic properties against bovine neutrophils. Further experiments are needed to evaluate the activity against other serotypes of *M. haemolytica*, in vivo studies and cell cytotoxicity assays.

## Supplementary Information

**Additional file 1. Microcin J25 characterization.** A) HPLC chromatogram of crude Microcin J25 (MccJ25) showing elution of MccJ25 at 20 min following acetonitrile/water gradient on a reversed-phase column. mAU is a symbol for the milli-absorbance unit. B) MALDI-TOF of pure MccJ25, purified by reversed-phase HPLC, showing [M + H] + peak (observed 2107.7 and calculated. 2107). Intensity is relative abundance or signal intensity of the ions and m/z is mass to charge ratio.

**Additional file 2. HBD3 10AA analogue characterization.** A) HPLC chromatogram of crude 10AA analogue showing elution of at 13.1 min following acetonitrile/water gradient on a reversed-phase column. mAU is a symbol for the milli-absorbance unit. Panel B shows MALDI-TOF of pure 10AA analogue, purified by reversed-phase HPLC, showing [M + H]^+^ peak (found 1282.6, calculated. 1283) (B). Intensity is relative abundance or signal intensity of the ions and m/z is mass to charge ratio.

**Additional file 3. HBD3 20AA analogue characterization.** (A) HPLC chromatogram of crude 20AA analogue showing elution at 35 min following acetonitrile/water gradient on a reversed-phase column. mAU is a symbol for the milli-absorbance unit. (B) MALDI-TOF of pure 20AA analogue, purified by reversed-phase HPLC, showing [M + H] ^+^ peak (found 2383.1, calculated. 2384). m/z is mass to charge ratio and Intensity is relative abundance or signal intensity of the ions.

**Additional file 4. HBD3 28AA analogue characterization.** Panels A shows MALDI-TOF of crude 28AA analogue, showing [M + H] ^+^ peak (found 3386.4, calculated. 3387). m/z is mass to charge ratio and Intensity is relative abundance or signal intensity of the ions.

**Additional file 5. Characterization of microbicidal activity of HBD3 28 AA analogue.** Colony forming units of *M. haemolytica*; that survived after incubation with 28AA analogue at concentration 50.0 μg/mL (A), 25.0 μg/mL (B), 12.5 μg/mL (C), 6.25 μg/mL (D), Negative control plate (E) and positive control plate (F).

**Additional file 6. Gradient elution scheme for 10AA and 20AA HBD3 analogues.**

**Additional file 7. Number of colony forming units after treatment with HBD3.**

**Additional file 8. Number of colony forming units after treatment with HBD3 10 AA analogue.**

**Additional file 9. Number of colony forming units after treatment with HBD3 20 AA analogue.**

**Additional file 10. Number of colony forming units after treatment with HBD3 28 AA analogue.**

**Additional file 11. Number of colony forming units after treatment with Mccj25.**

## Data Availability

The protocols and the data obtained from the experiments reported in the manuscript are included in the Additional tables and figures.
