# Supplementary figures and images for: Engineering and characterization of human β-defensin-3 and its analogues and microcin J25 peptides against Mannheimia haemolytica and bovine neutrophils
Source: Vet Res. 2021 Jun 10;52:83. doi: 10.1186/s13567-021-00956-4 (PMC8194028; doi:10.1186/s13567-021-00956-4)

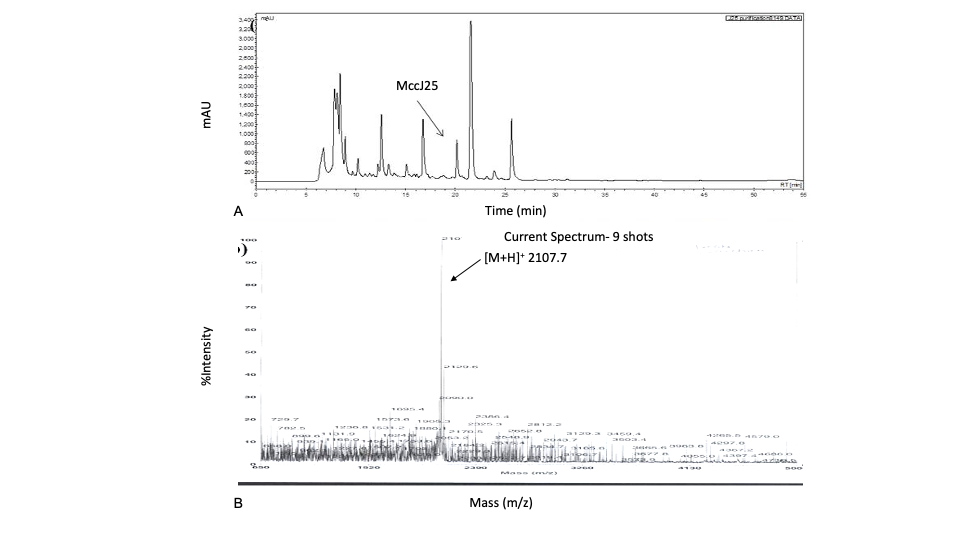

Supplement: Supplementary file 1 — Additional file 1. Microcin J25 characterization. A) HPLC chromatogram of crude Microcin J25 (MccJ25) showing elution of MccJ25 at 20 min following acetonitrile/water gradient on a reversed-phase column. mAU is a symbol for the milli-absorbance unit. B) MALDI-TOF of pure MccJ25, purified by reversed-phase HPLC, showing [M + H] + peak (observed 2107.7 and calculated. 2107). Intensity is relative abundance or signal intensity of the ions and m/z is mass to charge ratio. [file 13567_2021_956_MOESM1_ESM.png]

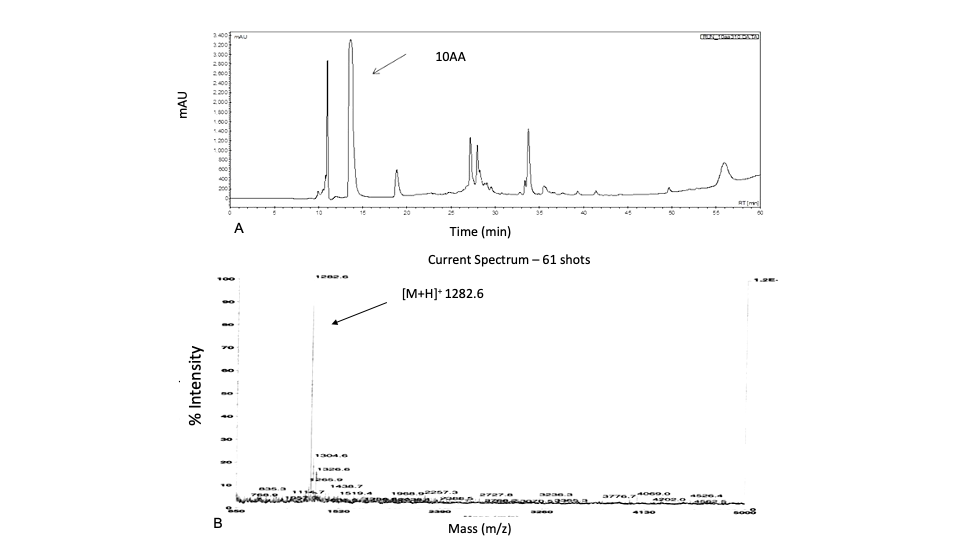

Supplement: Supplementary file 2 — Additional file 2. HBD3 10AA analogue characterization. A) HPLC chromatogram of crude 10AA analogue showing elution of at 13.1 min following acetonitrile/water gradient on a reversed-phase column. mAU is a symbol for the milli-absorbance unit. Panel B shows MALDI-TOF of pure 10AA analogue, purified by reversed-phase HPLC, showing [M + H]+ peak (found 1282.6, calculated. 1283) (B). Intensity is relative abundance or signal intensity of the ions and m/z is mass to charge ratio. [file 13567_2021_956_MOESM2_ESM.png]

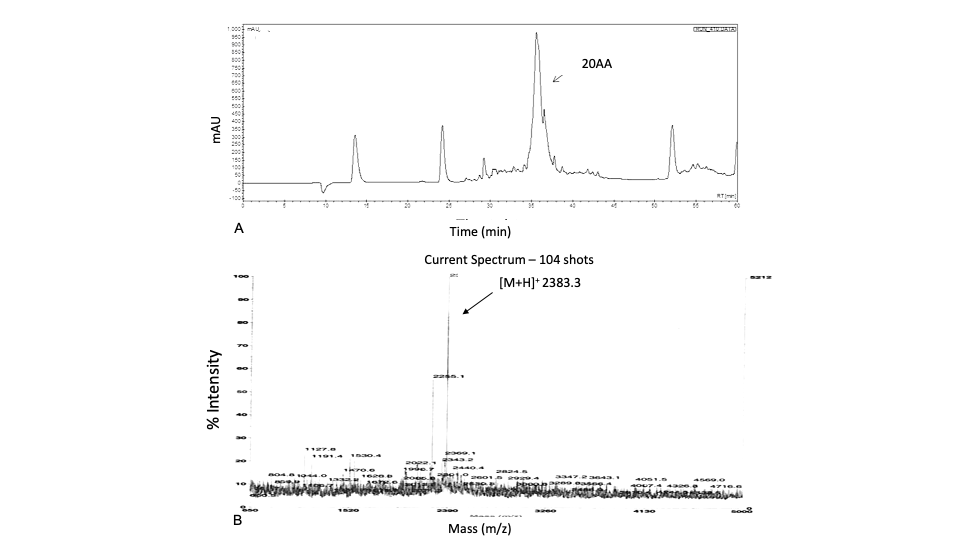

Supplement: Supplementary file 3 — Additional file 3. HBD3 20AA analogue characterization. (A) HPLC chromatogram of crude 20AA analogue showing elution at 35 min following acetonitrile/water gradient on a reversed-phase column. mAU is a symbol for the milli-absorbance unit. (B) MALDI-TOF of pure 20AA analogue, purified by reversed-phase HPLC, showing [M + H] + peak (found 2383.1, calculated. 2384). m/z is mass to charge ratio and Intensity is relative abundance or signal intensity of the ions. [file 13567_2021_956_MOESM3_ESM.png]

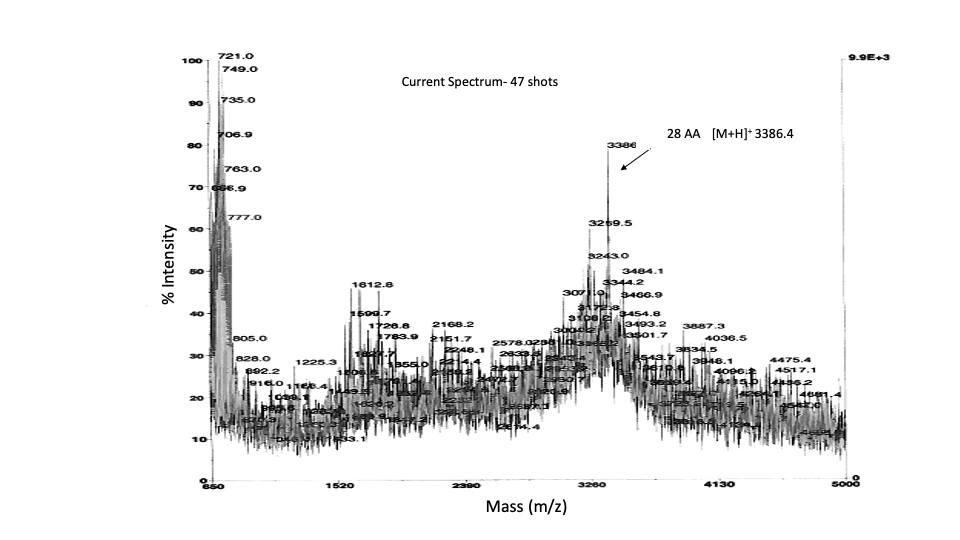

Supplement: Supplementary file 4 — Additional file 4. HBD3 28AA analogue characterization. Panels A shows MALDI-TOF of crude 28AA analogue, showing [M + H] + peak (found 3386.4, calculated. 3387). m/z is mass to charge ratio and Intensity is relative abundance or signal intensity of the ions. [file 13567_2021_956_MOESM4_ESM.png]

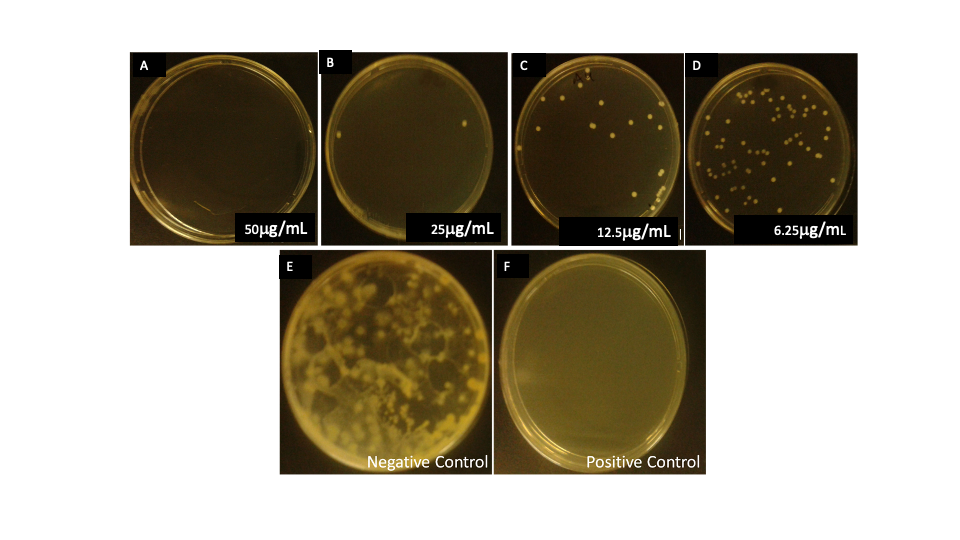

Supplement: Supplementary file 5 — Additional file 5. Characterization of microbicidal activity of HBD3 28 AA analogue. Colony forming units of M. haemolytica; that survived after incubation with 28AA analogue at concentration 50.0 μg/mL (A), 25.0 μg/mL (B), 12.5 μg/mL (C), 6.25 μg/mL (D), Negative control plate (E) and positive control plate (F). [file 13567_2021_956_MOESM5_ESM.png]
