# Supplementary material for: Engineering and characterization of human β-defensin-3 and its analogues and microcin J25 peptides against Mannheimia haemolytica and bovine neutrophils
Source: Vet Res. 2021 Jun 10;52:83. doi: 10.1186/s13567-021-00956-4 (PMC8194028; doi:10.1186/s13567-021-00956-4)
Supplement: Supplementary file 6 — Additional file 6. Gradient elution scheme for 10AA and 20AA HBD3 analogues. [file 13567_2021_956_MOESM6_ESM.docx]

| **Time (min)** | **Acetonitrile (%)** | **Water (%)** |
| --- | --- | --- |
| **0** | 10 | 90 |
| **30** | 17.5 | 82.5 |
| **60** | 35 | 65 |
