# Supplementary material for: Engineering and characterization of human β-defensin-3 and its analogues and microcin J25 peptides against Mannheimia haemolytica and bovine neutrophils
Source: Vet Res. 2021 Jun 10;52:83. doi: 10.1186/s13567-021-00956-4 (PMC8194028; doi:10.1186/s13567-021-00956-4)
Supplement: Supplementary file 7 — Additional file 7. Number of colony forming units after treatment with HBD3. [file 13567_2021_956_MOESM7_ESM.docx]

| Well No. | Peptide conc. μg/mL | No. of CFU | Well No. | Peptide conc. μg/mL | No. of CFU |
| --- | --- | --- | --- | --- | --- |
| A1 | 100.0 | 0 | B1 | 100.0 | 0 |
| A2 | 50.0 | 0 | B2 | 50.0 | 0 |
| A3 | 25.0 | 31 | B3 | 25.0 | 9 |
| A4 | 12.5 | 42 | B4 | 12.5 | 101 |
| A5 | 6.3 | 210 | B5 | 6.3 | 390 |
| A6 | 3.1 | >400 | B6 | 3.1 | >400 |
| A7 | 1.6 | >400 | B7 | 1.6 | >400 |
| A8 | 0.8 | >400 | B8 | 0.8 | >400 |
| A10 | - | 0 | B10 | - | 0 |
| A11 | - | 0 | B11 | - | 0 |
| A12 | - | >400 | B12 | - | >400 |

The tissue culture dishes were inoculated with 50 μL. solution from the respective wells and incubated for 24 h (This table shows data from one of three separate experiments). The final inoculum in this experiment was 5 × 10^5^ cfu/mL.
